# Genomic analysis of the initial dissemination of carbapenem-resistant *Klebsiella pneumoniae* clones in a tertiary hospital

**DOI:** 10.1099/mgen.0.001032

**Published:** 2023-06-05

**Authors:** Neris Garcia-Gonzalez, Begoña Fuster, Nuria Tormo, Carme Salvador, Concepcion Gimeno, Fernando Gonzalez-Candelas

**Affiliations:** ^1^​ Joint Research Unit 'Infection and Public Health', FISABIO-University of Valencia, Institute for Integrative Systems Biology (I2SysBio), Valencia, Spain; ^2^​ Microbiology Department, Valencia General University Hospital Consortium, Valencia, Spain; ^3^​ Faculty of Medicine, University of Valencia, Valencia, Spain; ^4^​ CIBER in Epidemiology and Public Health, Valencia, Spain

**Keywords:** *Klebsiella pneumoniae*, carbapenem resistance, plasmids, surveillance

## Abstract

Carbapenem-resistant *Klebsiella pneumoniae* is a major cause of hospital-acquired infections and the fastest-growing pathogen in Europe. Carbapenem resistance was detected at the Consorcio Hospital General Universitario de Valencia (CHGUV) in early 2015, and there has been a significant increase in carbapenem-resistant isolates since then. In this study, we collected carbapenem-resistant isolates from this hospital during the period of increase (from 2015 to 2019) and studied how *K. pneumoniae* carbapenem-resistant isolates emerged and spread in the hospital. A total of 225 isolates were subjected to whole-genome sequencing with Illumina NextSeq. We characterized the isolates by identifying lineages and antimicrobial resistance genes and plasmids, especially those related to reduced carbapenem susceptibility. Our findings show that the initial carbapenem resistance emergence and dissemination at the CHGUV occurred during a short period of 1 year. Furthermore, it was complex, involving six different lineages of types ST307, ST11, ST101 and ST437, different resistance-determinant factors, including OXA-48, NDM-1, NDM-23 and DHA-1, and different plasmids.

## Data Statement

All sequencing reads generated in this work have been deposited in the National Center for Biotechnology Information (NCBI) Sequence Read Archive (SRA) under BioProject accession number PRJEB37504. Refer to Table S2 (available in the online version of this article) for SRA accession numbers. Code and protocols have been provided within the article or through supplementary data files. Eight supplementary tables and six supplementary figures are available with the online version of this article.

## Introduction

*Klebsiella pneumoniae* belongs to the family *Enterobacteriaceae* and is a major cause of nosocomial and community-acquired infections that are particularly problematic in healthcare settings, seriously affecting neonatal and other intensive care units [1]. The main group of *K. pneumoniae* causing the most concern are the carbapenemase producers. They have expanded worldwide in the last decade and are included in the World Health Organization’s list of critical antibiotic-resistant pathogens for which new antibiotics are urgently needed [2]. Carbapenem-resistant *K. pneumoniae* (CRKp) is the most rapidly increasing pathogen in terms of the number of infections and the number of attributable deaths in Europe [3]. Carbapenemases can hydrolyze most of the commonly used beta-lactam antibiotics and are usually encoded in mobile elements such as plasmids [4]. In addition to carbapenemase genes, plasmids often carry resistance genes to other antibiotic families such as fluoroquinolones and aminoglycosides, which results in multidrug-resistant (MDR) strains. The combination of several resistance factors in the same strain makes these infections very difficult to treat [4]. Consequently, CRKp causes high mortality and morbidity, mainly due to the lack of therapeutic alternatives for these infections [5]. The most frequent carbapenemases in the family *Enterobacteriaceae *, and also in *K. pneumoniae,* are KPC, VIM, NDM, IMP and OXA-48 [6]. In Spain, *K. pneumoniae* is the species carrying the most carbapenemase genes, mainly *bla*_OXA-48_ [7–9]. It has been shown that *bla*_OXA-48_ genes are quite versatile and they have been associated with different sequence types (STs), such as ST15, ST11, ST405 and ST307 clones [7, 8]. Other carbapenem resistance genes, such as *bla*_VIM_ or *bla*_KPC_, have been detected in Spain, but at much lower frequencies [9, 10].

The Consorcio Hospital General Universitario de Valencia (CHGUV) is a tertiary level hospital with a reference population of almost 360 000 inhabitants in Valencia, Spain. The first cases of carbapenemase-producing *Enterobacteriaceae* – mainly *K. pneumoniae* carrying *bla*_OXA-48_ – were detected in 2014. Since then, the number of such isolates has risen dramatically in this healthcare setting, especially in 2016, when several outbreaks of carbapenem-producing *K. pneumoniae* were detected [11]. Here, we have analysed *K. pneumoniae* isolates non-susceptible to carbapenems collected during the first 4 years after their detection at this hospital. We applied whole-genome sequencing (WGS) to characterize these multidrug-resistant isolates and their plasmids, and to perform genomic epidemiology analyses.

## Methods

### Isolate selection and susceptibility testing

Carbapenem-resistant strains of *K. pneumoniae* isolated in routine screenings at the CHGUV between January 2015 and December 2018 were included in the study. The only inclusion criterion was non-susceptibility to any carbapenem (ertapenem, imipenem, or meropenem) regardless of the sample type. A total of 257 strains were obtained from clinical samples from different hospital wards, including active surveillance cultures from the intensive care unit (pharyngeal and axillary/faecal carriages). Forty-one carbapenem-susceptible and extended-spectrum beta-lactamase (ESBL)-producing contemporary *K. pneumoniae* strains were also included in the study as controls.

Antimicrobial susceptibility testing was performed by broth microdilution using the automated system MicroScan WalkAway (Becton Dickinson). The minimal inhibitory concentrations (MICs) of ampicillin, amoxicillin–clavulanate, amikacin, cefepime, cefotaxime, cefoxitin, ceftazidime, cefuroxime, ciprofloxacin, colistin, ertapenem, gentamicin, imipenem, norfloxacin, piperacillin–tazobactam, tigecycline, trimethoprim–sulfamethoxazole, meropenem and levofloxacin were determined. The antibiotics being tested varied depending on the origin of the sample. Susceptibility breakpoints were interpreted according to the recommendations of the Clinical and Laboratory Standards Institute (CLSI) [12]. We adopted the definitions of MDR and extensively drug-resistant (XDR) from Magiorakos *et al*. [13].

### WGS and comparative analyses

The selected isolates were plated on blood agar (Becton Dickinson) and incubated overnight at 37 °C. Genomic DNA was extracted using the automated system MagCore (RBD Science). The DNA libraries were prepared using the Nextera XT sample preparation method. Isolates were sequenced using an Illumina NextSeq (San Diego, CA, USA) platform with 150 bp paired-end reads.

FastQC v0.11.9 [14] and MultiQC v1.11 [15] were used to assess the quality of the reads from every sequencing experiment. PrinSEQ v0.20.4 [16] was used to clean, filter and trim the raw reads, removing reads with a mean quality <28 and trimming the 10 left positions.

Species confirmation, sequence typing and identification of resistance genes and genes encoding virulence determinants were performed using Kleborate v2.1.0 [17] and Kaptive [18, 19]. The identification of plasmid incompatibility groups was performed using PlasmidFinder v 2.1 and its built-in *Enterobacteriaceae* database (downloaded 15 November 2021) [20].

Draft genome assemblies were generated using SPAdes v3.14.1 with default settings [21]. The quality of the assemblies was assessed using QUAST v5.0.2 [22]. Isolates with more than 1000 contigs detected as weak or non-strict *K. pneumoniae* species with Kleborate were removed from subsequent analyses due to suspected contamination or sequencing errors. Contigs were annotated with PROKKA v1.14.6 [23].

For comparative analysis, all *K. pneumoniae* genome assemblies labelled as (‘*Klebsiella pneumoniae’* AND ‘Spain’) in the European Nucleotide Archive (ENA) (accessed on 23 May 2022) were included in the analysis. We used the ncbi-genome-download tools v0.3.1 (https://github.com/kblin/ncbi-genome-download) to download the assemblies and the E-utilities commands to extract the metadata. We only kept reference genomes with no QC warning in Kleborate.

Genetic relatedness among isolates was evaluated using core genome phylogenetic trees. We used panaroo v1.2.10 [24] with the strict mode to construct relaxed core genomes at 90 % unless indicated otherwise. Each gene family was realigned with mafft v7.490 with the --adjust-direction option [25]. The concatenation of genes for each sample was made with AMAS.py concat [26]. The core genome alignment was trimmed using ClipKit v1.3.0 [27] with the kpic-gappy mode and used to infer a maximum-likelihood (ML) phylogenetic tree with IQTREE v2.0.7 [28] with the option -TEST to find the best fitting substitution model. Ultrafast bootstrap branch supports were assessed after 1000 pseudorandom replicates [29]. The ML tree was visualized using ITOL v6 [30]. We used snp-sites v2.5.1 [31] to extract SNP positions from the alignment. The same procedure for obtaining the core genome and an ML tree was applied to the subset of samples corresponding to each of the four major STs found in this work.

We searched the genomes for genetic determinants that could be relevant to carbapenem resistance and divided the entire isolate collection into four Carba-R groups. Group 1 included isolates carrying a known carbapenemase gene regardless of other mechanisms. Group 2 encompassed isolates carrying any *ampC* gene. Group 3 included isolates with truncated or absent porins. Group 4 included carbapenem-resistant strains with no described resistance mechanism to these antimicrobial agents.

To study the variants and mobility of possible plasmids with carbapenem resistance genes, we performed a mapping analysis using snippy v4.6.0 [32] with a minimum coverage of four reads, a minimum fraction of 70% and a minimum quality of 60. For the OXA48-producing isolates, we used the reference plasmid NC_019154. This plasmid showed a high similarity in terms of primary sequence and structure to those of this work. For the NDM-producing strains, we used plasmid NZ_CAKLAW010000003 extracted from the GCF_920939505 genome, a closed genome sequence from one of the samples included in this study [33]. To minimize the effect of spurious SNPs due to mapping errors, we checked individual variant positions with IGV [34]. ML phylogenetic trees for both plasmid alignments were obtained with IQTREE v2.0.7 [28] with the -cbz option.

## Results

### Isolates, clinical data and WGS

Initially, we selected 257 *K *. *pneumoniae* isolates non-susceptible to carbapenem for analysis, 183 of which were finally prepared for WGS. The remaining isolates were discarded due to lack of growth, or contamination of the culture plates. The 41 carbapenem-susceptible control strains were subjected to complete genome sequencing.

Patients were mostly elderly, with a median age of 74 years (range 23–94). Regarding isolation sources, the most frequent ones were those from urine (98 samples, 44 %), followed by axillary/faecal carriage (68 samples, 30 %) (Table S1, available in the online version of this article).

For the 224 sequenced samples, an average of 1 981 172 reads per sample was obtained, with a maximum of 4 139 494 and a minimum of 849 554 reads. After quality evaluation with FastQC, all the reads showed a Phred quality score >30 in all the positions. Samples were assembled into an average of 110 contigs (range 43–427). The average N50 value was 175 192.28 (range 46 982–368 804). The reconstructed whole-genome assemblies ranged between 5.3 and 5.5 Mb in length, and the GC percentage varied between 56.4 and 57.63 % (Table S2).

Regarding the isolates downloaded from GenBank, we included 360 assemblies in the analysis. Isolates were collected between 2008 and 2018. However, most of those isolates were collected in a multi-regional study in 2018 [35] (Table S3).

### Carbapenem non-susceptibility is related to four major STs, and OXA-48 and NDM carbapenemases

**Fig. 1.**
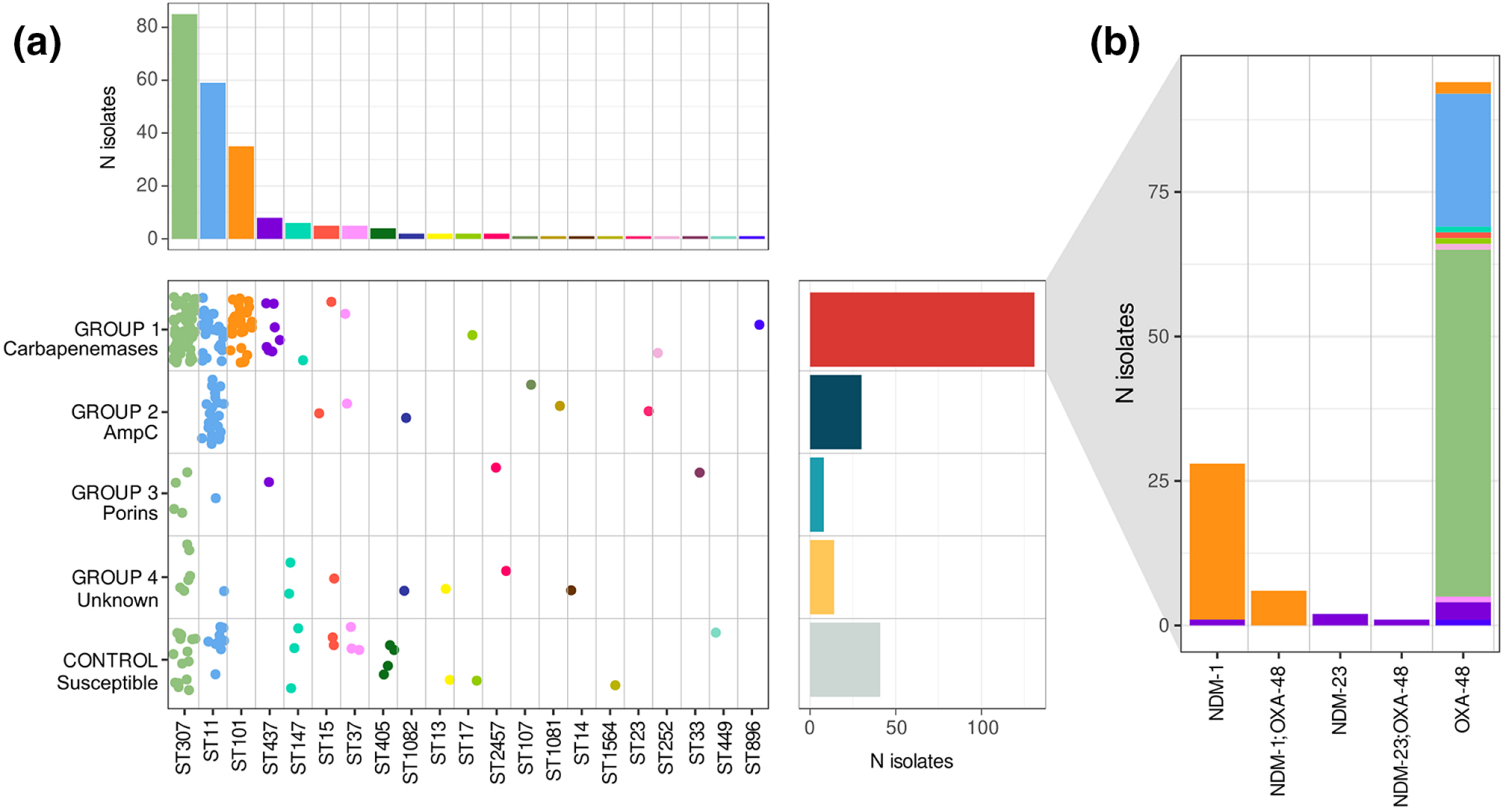
**(a**) Isolate distribution in Carba-R groups and STs. Dots in the central plot and bars in the upper barplot are coloured by ST, while bars in the right barplot are coloured by Carba-R group. (**b**) Distribution of the different Carba-R genes found in group 1. Bars are coloured by ST.

*In silico* typing identified 21 different known STs, among which ST307, ST11, ST101 and ST437 were the most prevalent ones and accounted for 88.5 % (162/183) of all the CRKp (Fig. 1a). The 183 CRKp isolates were classified into four different Carba-R groups by the nature of their non-susceptibility to carbapenems (Fig. 1a).

Group 1 encompassed the largest carbapenem resistance group and included 131 isolates with acquired carbapenemases. It was mainly associated with the most prevalent STs: ST307, ST11, ST101 and ST437. Two different carbapenemase genes were detected in group 1: *bla*_OXA-48_, in 101 isolates (mainly ST307 and ST11), and 2 variants of *bla*_NDM_ genes, *bla*_NDM-1_ and *bla*_NDM-23_, in 34 and 3 isolates, respectively. While *bla*_NDM-1_ was found in ST101 and ST437, *bla*_NDM-23_ was only found in ST437. Remarkably, we found seven isolates from the two STs, ST101 and ST437, harbouring both *bla*_OXA-48_ and *bla*_NDM_ genes (Fig. 1b).

Group 2 included carbapenem-resistant strains due to the acquisition of an *ampC* gene. This group included 30 isolates of ST11. AmpC was found to be mediated only by DHA-1.

Group 3 included missing or truncated porins (eight isolates) and was related to five different STs. Porin truncations were only detected in the *Ompk36* gene. We found one isolate with a Gly134Asp135 duplication in loop 3 (OmpK36GD). The antibiotic resistance levels produced by this duplication are similar to those produced by the loss or truncation of the porin [36].

Group 4 included 14 isolates for which none of the previously described mechanisms could explain low carbapenem susceptibility. In 11 of these isolates, belonging to 8 different STs, resistance to carbapenems might be attributed to the presence of ESBL genes (11/14), especially *bla*_CTX-M-15_ (8/11).

Carba-R groups differed in their population diversities. Although some isolates from major lineages, such as ST101 and ST437, were restricted to a single group, those in other lineages, such as ST307 and ST11, were found in different subgroups, indicating possible different populations. Conversely, some Carba-R groups, such as 3 and 4, were highly diverse, presenting several different STs, with no single lineage associated with them.

### Carbapenem-resistant strains in the CHGUV were disseminated by six lineages

**Fig. 2.**
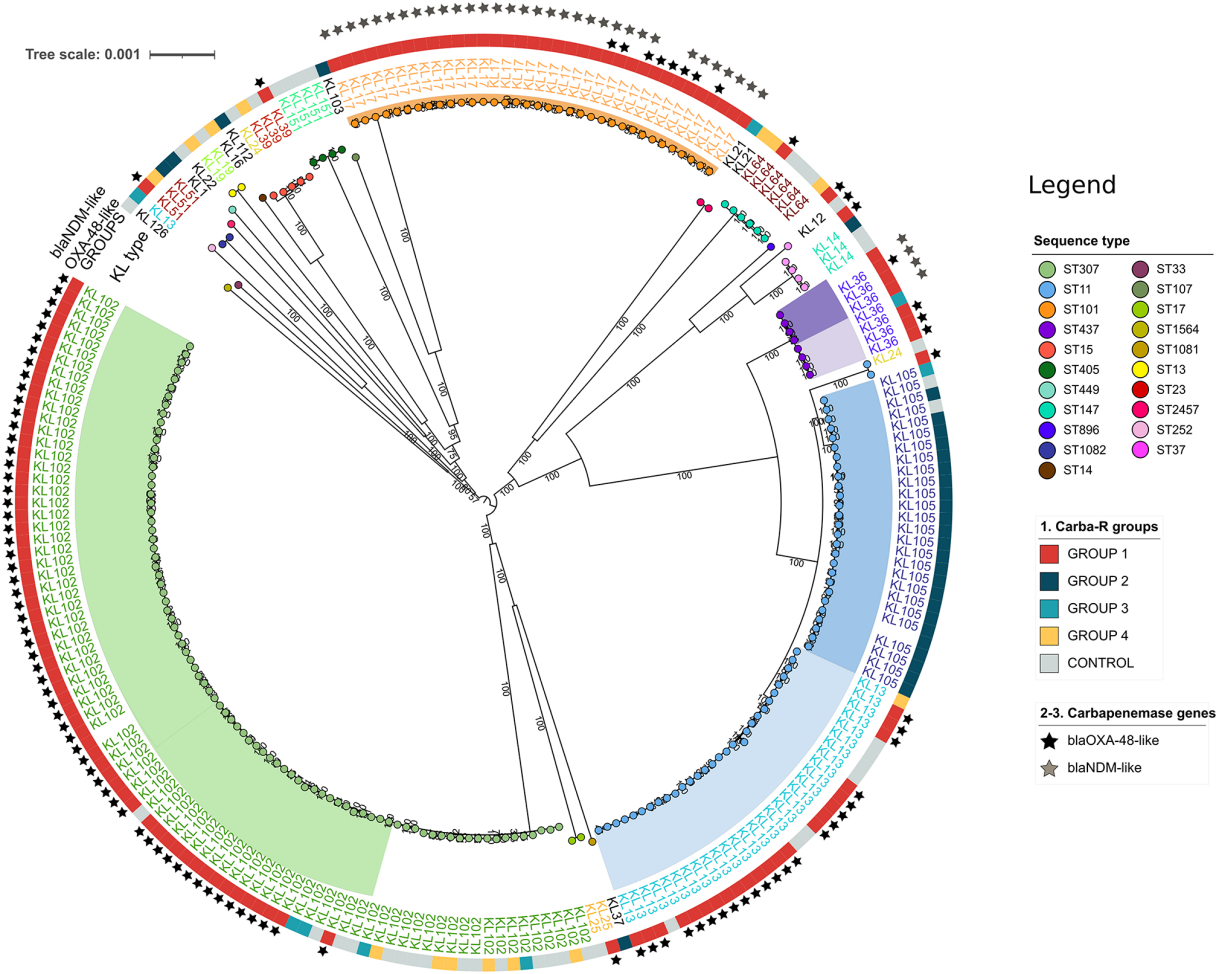
Maximum-likelihood phylogenetic tree of the *K. pneumoniae* isolates collected at the CHGUV from 2015 to 2018. The tree was constructed from the core genome (90%) alignment (3 570 188 bp) of the 224 isolates. STs, capsular loci, Carba-R groups and the presence of *bla*_OXA-48_ and *bla*_NDM_ genes are shown.

In mid-2015, a rapid increase of carbapenem non-susceptible *K. pneumoniae* isolates was observed at the hospital (Fig. S1). The ‘outbreak’ was mostly associated with the expansion of certain STs. To understand the relationships between the STs found in the CHGUV, we obtained the core genome and derived the corresponding ML phylogenetic tree (Fig. 2). The phylogeny, capsular types and antimicrobial resistance genes (ARGs) revealed that ST307, ST11 and ST437 could be divided into different lineages. To facilitate the ensuing description of the sublineages, we named them following their capsular types and ARGs. The ST307 was divided into two different clades, ST307-OXA48 (66 isolates) and ST307-CONTROLS (13 isolates). The ST11 was divided into 3 lineages: ST11-KL13 (30 isolates), ST11-KL105 (27 isolates) and ST11-KL24 (2 isolates). The ST437 clade was split into two clades, ST437-OXA48 (four isolates) and ST437-NDM (four isolates). Finally, ST101 was retained as a single lineage (34 isolates).

The genetic diversity within lineages was much lower than that between lineages (Fig. S3, Tables S5–8), likely supporting being different clades. This indicates that each sublineage corresponds to a single dissemination following a clonal expansion and not multiple, independent introductions and subsequent spreads. In fact, some isolates were highly similar (pairwise SNP distances <5), possibly belonging to the same transmission chains.

### Antimicrobial susceptibility of Carba-R groups and sublineages and association with other genetic features (ARGs and plasmids)

All the isolates, including the controls, were MDR strains, as they were non-susceptible to at least one drug in three or more antimicrobial categories (Table S1). In general, all the isolates were resistant to penicillins, cephalosporins and quinolones, and most of them were also resistant to cotrimoxazole and ertapenem. Colistin was the antibiotic with the highest susceptibility rates (69.5 %, MICs lower than 2 mg l^−1^), followed by tigecycline (69.5 %).

**Fig. 3.**
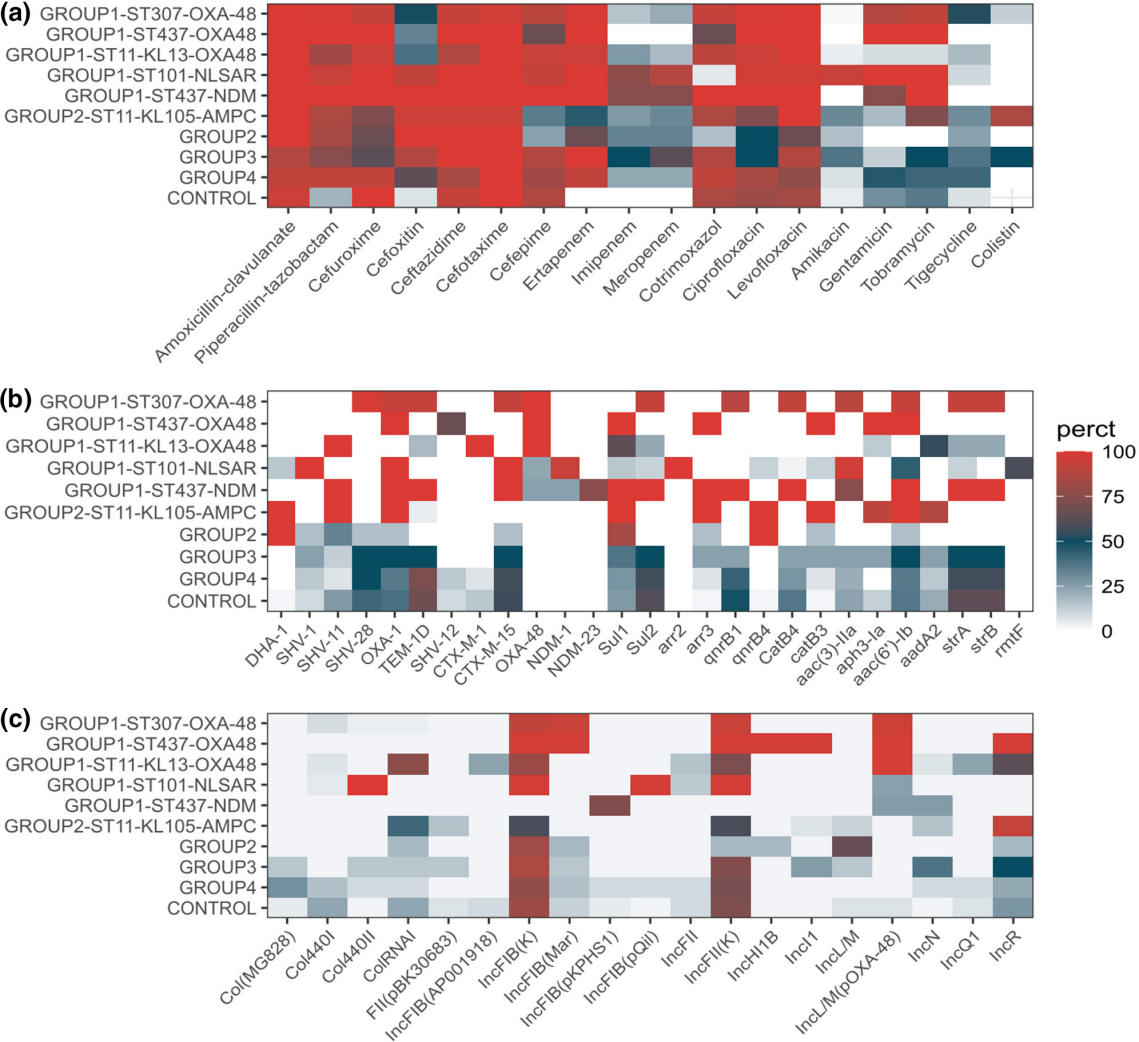
The proportion of isolates in each Carba-R group and sublineage that (a) showed phenotypic resistance to each antimicrobial tested, (**b**) carried the antimicrobial resistance genes identified and (c) harboured any of the plasmid incompatibility groups detected.

We observed an association between Carba-R groups and sublineages, susceptibility to antimicrobials and ARG repertoire (Fig. 3, Table S2).

The most prevalent carbapenemase gene was *bla*_OXA-48_, which was linked to lineages ST307-OXA48, ST11-KL13 and ST437-OXA-48.

Isolates in lineage ST307-OXA48 were resistant to most of the antibiotics tested and had a high proportion of tigecycline resistance (52.5 %). Almost all the isolates in this lineage carried *bla*_SHV-28_, *bla*_TEM-1D_, *bla*_OXA-1_, *bla*_CTX-M-15_, *sul2*, *qnrB1*, *catB4*, *aac(3)-IIa*, *acc(6)-Ib-cr*, *strA* and *strB* genes.

Isolates in lineage ST437-OXA-48 had a similar antimicrobial susceptibility profile to those in the ST307-OXA48, yet the ARGs present were different, as they carried *bla*_OXA-1_, *bla*_SHV-12_, *bla*_OXA-48_, *sul1*, arr3, CatB3, *aac6′-Ib* and *aph(3)-Ia*.

Isolates in lineage ST11-KL13 showed a very different susceptibility pattern, with a lower load of AMR genes than ST307-OXA48, reflected in lower resistance levels to aminoglycosides and tigecycline. The genes found to be present in all of the isolates of the clade were *bla*_SHV-11_, *bla*_CTX-M-1_, *bla*_OXA-48_ and, in some isolates, *bla*_TEM-1D_ (18.1 %), *Sul1* (63.3 %), *Sul2* (22.7 %) and *aadA*2 (54.5 %).

These three lineages had different plasmids but share the presence of the IncL/M(pOXA-48) plasmid, highly associated with *bla*_OXA-48_ genes (Fig. 3c).

The other carbapenemases found, NDM-1 and NDM-23, were associated with group 1 lineages ST101 and ST437-NDM. Isolates belonging to the ST101 lineage were resistant to almost all the antibiotics tested except colistin, cotrimoxazole and tigecycline, due to the combined effect of the genes *bla*_NDM-1_, *bla*_SHV-1_, *bla*_OXA-1_, *bla*_CTX-M-15_, *arr-2*, *aac(3)-IIa* and, in some cases, also *bla*_OXA-4_8 (23.5 %) and *acc(6)-Ib* (44.1 %). Moreover, this lineage was the only one carrying *rmtF* (58.8 %), which explains the amikacin resistance that was hardly seen in the other lineages. Although this lineage was largely associated with *bla*_NDM_ genes (94.1 %), we found some isolates that had lost the *bla*_NDM_ gene but had kept *bla*_OXA-48_ (5.8 %).

Lineage ST437-NDM isolates had a similar antimicrobial susceptibility profile to those of ST101, with the difference that they were resistant to cotrimoxazole and susceptible to amikacin. Isolates in ST437-NDM had a large number of AMR genes consisting of *bla*_SHV-11_, *bla*_OXA-1_, *bla*_TEM1-D_, *bla*_CTX-M-15_, sul1, sul2, *arr-3*, qnrB1, CaB4, *acc(6)-I, strA* and *strB*. All the isolates in this lineage carried *bla*_NDM_ variants, 25 % carried *bla*_NDM-1_ and 75 % *bla*_NDM-23_. Moreover, 25 % of those isolates also carried *bla*_OXA-48_. As expected, only those lineages that carried *bla*_NDM_ genes were resistant to the three carbapenems tested (ertapenem, meropenem and imipenem).

Although lineages ST101 and ST437-NDM were associated with NDM carbapenemases, they carried different plasmids, and none of them were found in both lineages (Fig. 3c), suggesting that those carbapenemases are encoded in different plasmids in these lineages.

Group 2 was mainly formed by isolates of lineage ST11-KL105, which included almost all *ampC* producers. This lineage showed lower levels of resistance than the groups mentioned above, with more susceptibility to cephalosporins and cotrimoxazole. Almost all the isolates of this group carried *bla*_DHA-1_, *bla*_SHV-11_, *bla*_OXA-1_, *sul1*, *arr3*, *qnrB4*, *catB3*, *aac6′-Ib, aph(3)-Ia* and *aadA2* genes. The remaining isolates of group 2 belonged to several different lineages, and this is reflected in the variation in the presence of ARGs. The only genes present in all the isolates were *bla*_DHA-1_ and *qnrB4*.

Isolates in group 3 (defective porins) and group 4 (unknown mechanism) and controls showed a highly diverse composition of STs (Fig. 1a) and were very variable in their antimicrobial susceptibility profiles and ARG repertoires. No common ARG was found to be associated with any of these groups. Nevertheless, we found some enrichment in *bla*_TEM-1D_, *bla*_OXA-1_, *bla*_CTX-M-15_, *sul2*, *qnrB1*, *aac(3)-IIa*, *acc(6)-Ib*, *strA* and *strB*. Isolates in group 3 (defective porins) and controls showed more susceptibility to beta-lactams than any other group.

### Most lineages were also found in other Spanish regions

**Fig. 4.**
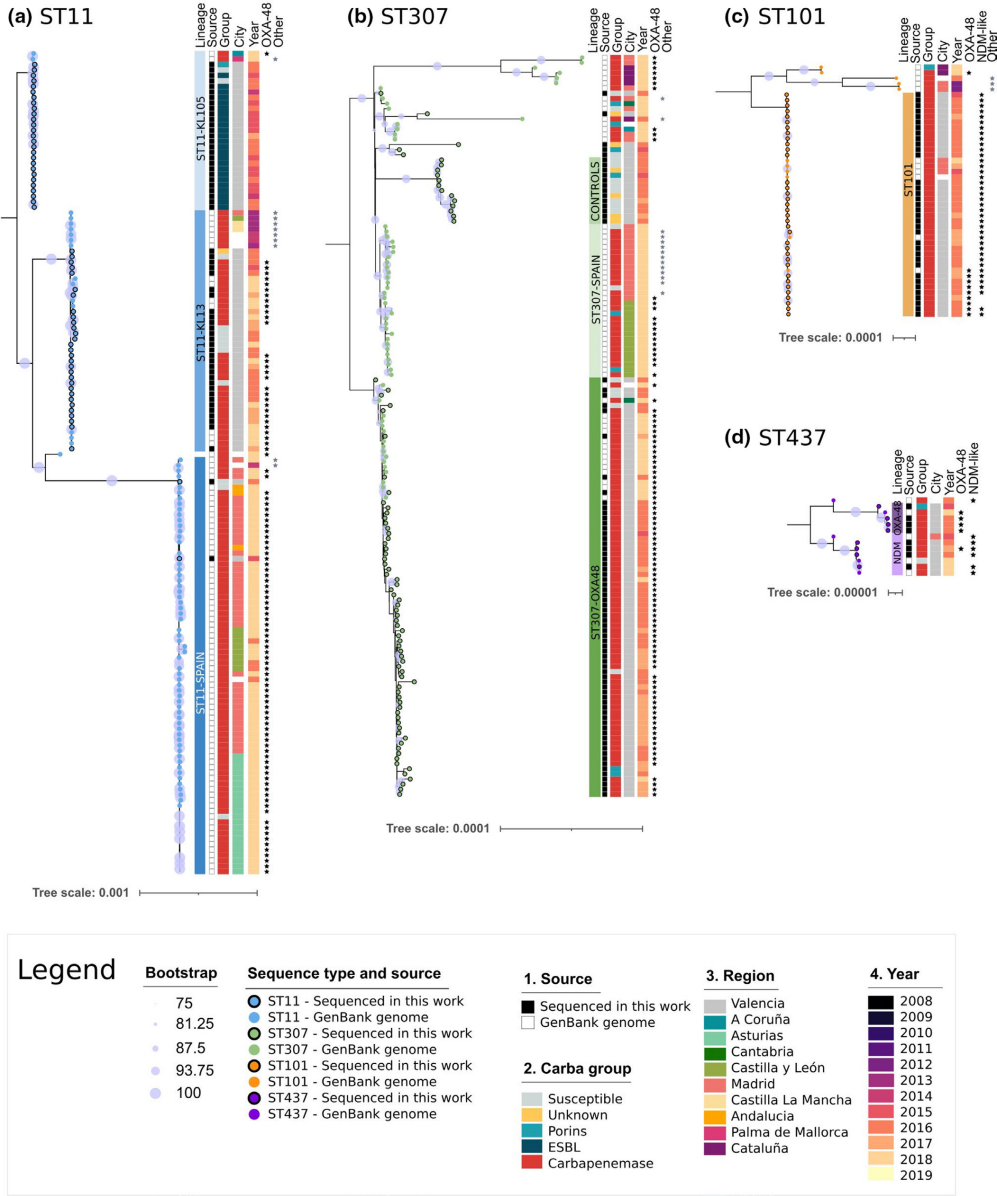
Maximum-likelihood phylogenetic trees (and length of the alignments used) for the (a) ST11 (4 081 060 bp), (**b**) ST307 (4 461 655 bp), (**c**) ST101 (4 278 965 bp) and (d) ST437 (4 346 386 bp) isolates from the CHGUV and downloaded from GenBank with Spanish origin.

To better understand and contextualize how these lineages emerged in the CHGUV, we added Spanish genomes (*n*=360) to the global analysis (Fig. S2, Table S4) and to those of each major ST (Fig. 4). The two major STs in the CHGUV, ST11 and ST307, were also frequently reported in Spain, whereas the other major lineages found in the CHGUV were seldom found in other locations. Moreover, lineages ST147, ST405, ST15, ST392 and ST512, which were found to be frequently reported in Spain, were not detected in the CHGUV.

For lineages ST11-KL105, ST11-KL13, ST101 and ST437-NDM, there were related isolates from other Spanish regions (Fig. 4). Lineage ST11-KL105 had related isolates from 2018 collected in northwestern Spain (Ferrol) and the Balearic Islands (Palma de Mallorca). These isolates did not carry *bla*_DHA1_, but they carried *bla*_OXA-48_ and *bla*_VIM-1_. However, the clade was very homogeneous, with an average of 39.6 pairwise SNPs (range 1–119). Related isolates of clade ST11-KL13 were collected in 2013–2014 from the centre of Spain (Guadalajara, Toledo, Madrid and Ciudad Real) and in 2018 from other Valencian hospitals. However, the samples from the centre of Spain carried the *bla*_KPC-2_ gene but not the *bla*_OXA-48_ gene, as did the ST11-KL13 isolates collected in the CHGUV and other Valencian hospitals. Nevertheless, the clade had a low diversity (71.8 pairwise SNPs on average, range 0–192). The other ST11 Spanish samples fell into a new clade, ST11-SPAIN, with most isolates carrying a *bla*_OXA-48_ and collected in 2018 throughout Spain (Madrid, Guadalajara, Cadiz, and Santander). Only two isolates found in the CHGUV fell into this clade. The three ST11 clades were very distant from each other (>2000 SNPs), suggesting three different introductions in Spain (Table S5, Fig. S3).

Lineage ST101 included three genomes collected in Madrid between 2015 and 2018 and in Valencia in 2017. The average pairwise SNPs within the clade was 14.9 (range 0–65) in 4 years, suggesting a rapid dispersion of the founder clone (Table S6).

ST437-OXA48 only included isolates sequenced from the CHGUV and other Valencian hospitals and was a remarkably homogeneous clade (average 10.3 pairwise SNPs, range 0–21) (Table S7).

ST437-NDM had a basal genome collected in 2015 in Madrid, but it was phylogenetically distant from the rest of the clade (64.1 pairwise SNPs on average) (Table S7).

Some database genomes fell into the ST307-OXA48 clade. However, all of them (except one from Oviedo) were from Valencia. Spanish genomes with *bla*_OXA-48_ and *bla*_KPC-3_ from Madrid and Guadalajara formed a new clade (ST307-SPAIN), while other isolates collected throughout the Spanish territory (including Valencia) did not fall into any lineage. The ST307-OXA48 clade (including the database genomes) maintained a low diversity (average of 25.2 pairwise SNPs, range 0–102), meaning that all those genomes likely belonged to the same clonal expansion group. ST307-SPAIN isolates were more similar to the ST307-OXA48 clade than to the controls of the same hospital (201 vs 349 SNPs on average, respectively). This suggests that the origin of the ST307-OXA48 was not in the controls collected in the hospital (Table S8, Fig. S3).

### *bla*_OXA-48_ and *bla*_NDM_ genes were disseminating in three and two different plasmids, respectively

**Fig. 5.**
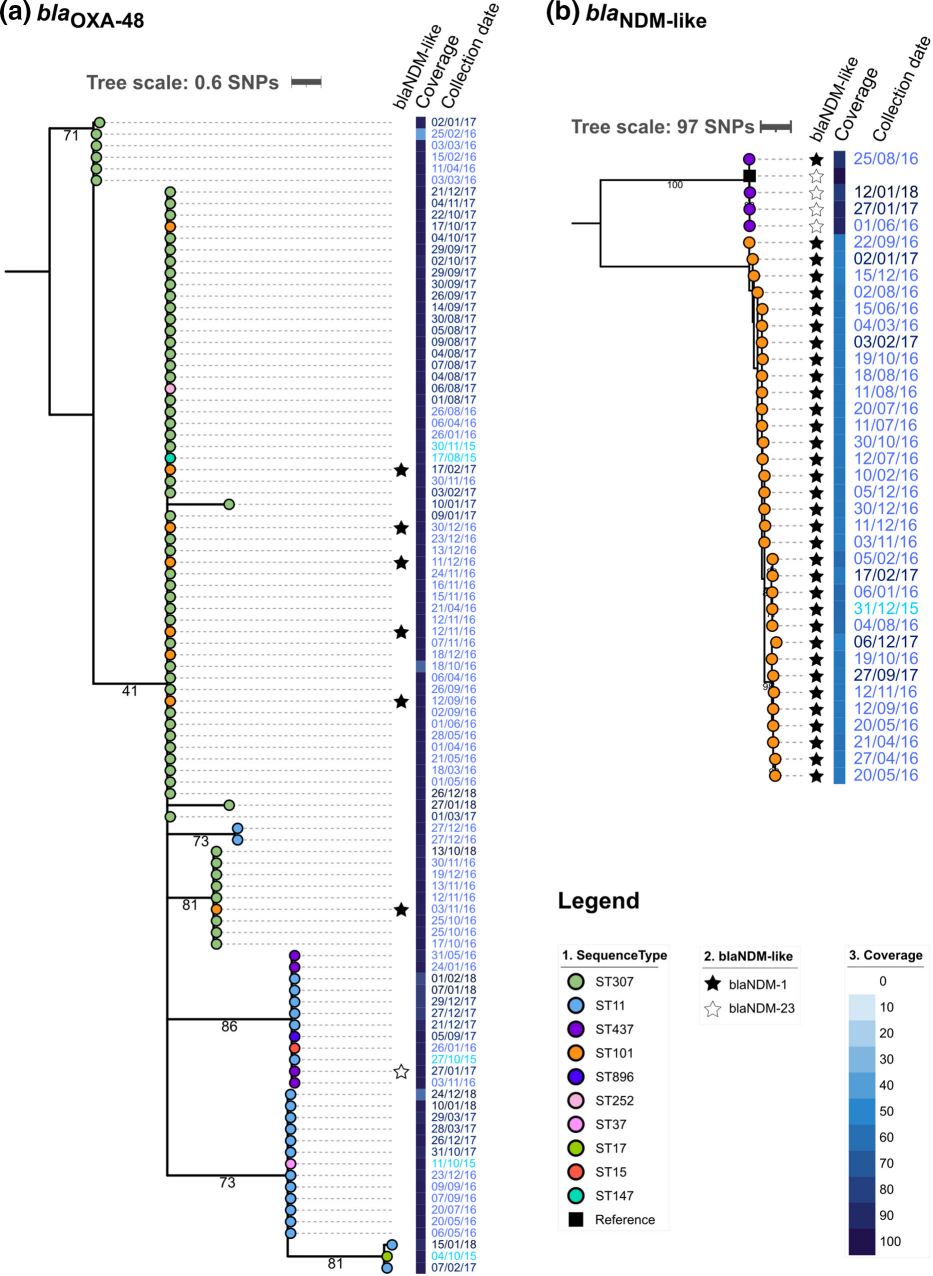
Maximum-likelihood phylogenetic trees for plasmids carrying carbapenemase genes (a) *bla*_OXA-48_ (61 881 bp) and (b) *bla*_NDM_-like (97 784 bp).

To understand the variability and dissemination of the different carbapenemase genes in the different lineages, we studied the plasmids carrying *bla*_OXA-48_ and *bla*_NDM_ genes. To study the spread of *bla*_OXA-48_ in the hospital, we obtained an ML tree from the core alignment of reads mapped to the reference plasmid NC_019154. This resulted in a 61 kb alignment with a median mapping coverage of 96 % (range 30–96 %) of the total plasmid length. Two isolates, 33KP-HG and 66KP-HG, mapped only 30 % of the plasmid length. These samples had the *bla*_OXA-48_ as an integron or inserted in a different plasmid backbone and thus were removed from subsequent analyses. The phylogeny of the *bla*_OXA-48_ plasmid (Fig. 5a) showed that at least three different plasmids or variants of the plasmid appeared almost simultaneously and coexisted at the hospital in several distinct lineages. We only considered as plasmid variants those that differed in at least two shared SNPs from any other variant (Fig. S5). We observed several cases of interlineage transfer of the plasmid within the different variants. Nevertheless, one variant was mainly associated with lineages ST307 and ST101, another variant was mainly associated with the ST11-KL13 lineage, and the third variant was associated with lineages ST11-KL13 and ST437-OXA48.

To understand the dissemination of the NDM plasmid, we mapped the sequencing reads of the samples to an NDM plasmid (NZ_CAKLAW010000003) collected at the CHGUV and obtained the ML tree shown in Fig. 5b. *bla*_NDM_ genes were detected in two lineages, ST101 and ST437-NDM. All the isolates in the ST437-NDM lineage mapped with more than 80 % coverage to the reference plasmid, whereas isolates in the ST101 lineage barely reached a coverage of 60 %. Based on the differences on the coverage and the number of SNPs between both lineages (Fig. S6), ST101 and ST437-NDM, we inferred that they hosted different NDM plasmids, although these two lineages appeared in the hospital simultaneously.

### The emergence of carbapenem resistance in the hospital was associated with travel and dissemination from other Spanish regions

**Fig. 6.**
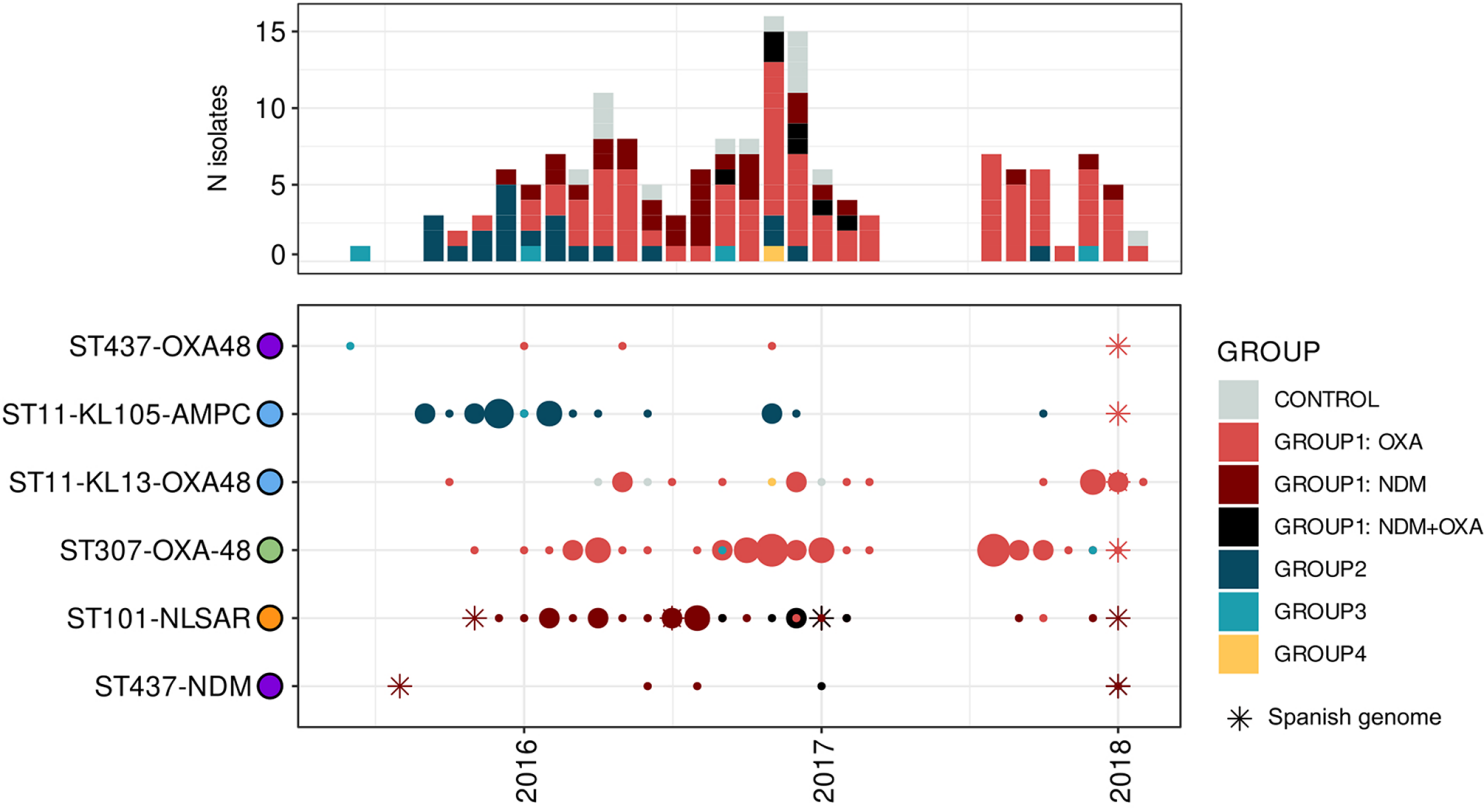
Distribution of Carba-R groups in each lineage throughout the sample collection period.

The lineages found in the CHGUV were detected almost simultaneously, but they dispersed differently through time. In the initial phase, before 2016, when the rise of isolates non-susceptible to carbapenem was observed, the major source of non-susceptibility to carbapenem was found to be the AmpC-producing ST11-KL105 lineage, along with occasional cases of isolates with truncated porins (Fig. 6). At some point in 2016, the AmpC-producing ST11-KL105 lineage was replaced by five different lineages that included two NDM-like-producing lineages, ST101 and ST437-NDM, and three OXA-48-producing lineages, ST437-OXA-48, ST307 and ST11-KL13.

For both the ST437-NDM and ST101 lineages, we found previous genomes in Madrid in 2015 that linked these cases to an already reported transmission chain [37]. Moreover, from epidemiological information, we could identify that the index patient in Madrid was the same as in the CHGUV. In both hospitals, both lineages entered simultaneously in the same patient, whose case was also related to travel. This patient was admitted to the Madrid hospital on his way home to Valencia from Pakistan, and a few months later he was transferred to the CHGUV with the infection [37]. Lineage ST11-KL13 has basal strains collected years before, from 2013 to 2014, in the centre of Spain, which indicates that this lineage was probably disseminated from other Spanish regions to the CHGUV. The ST307 lineage and ST11-KL105 show patterns of possible dissemination throughout the country, but their emergence in the CHGUV cannot be ascertained with the available data.

## Discussion

Currently, CRKp is a major contributor to antibiotic resistance in hospital settings [9]. Nevertheless, little is known at the genomic level about the initial colonization and subsequent dispersion of these isolates in hospitals. Most studies have analysed the specific bacterial clone and plasmid(s) responsible for the first cases detected [38]. Extending these analyses beyond the initial period and complete genome analysis of the corresponding isolates allows a better characterization of the colonizing strains and genes, revealing the relationships among isolates. These can then be used to establish the relative contributions of new introductions and the local spread of resistance genes and their mobilization vehicles in previously established strains and species. This information is crucial and, coupled with active genomic surveillance of resistance, may provide hints on how to prevent and control new entries and the spread of resistance determinants in the future.

This study describes the initial colonization of carbapenem-resistant *K. pneumoniae* isolates in a hospital in Valencia (Spain). A total of 224 genomes were sequenced. All the isolates were MDR and showed phenotypic resistance to a variety of antibiotics, including carbapenems, other beta-lactams, aminoglycosides and quinolones. The initial cases of CRKp in the CHGUV were mainly due to non-carbapenemase-producing *K. pneumoniae* strains. We found that the very early cases were related to the presence of *ampC* and truncated porins, while only a few were related to carbapenemase enzymes (Fig. 1). The AmpC mechanism was mediated by *bla*_DHA-1_ genes and disseminated by the ST11-KL105 lineage. The mutated porins were mainly due to the truncation of the Ompk36 porin. Nevertheless, other mutations, such as OmpK36GD, were also found. Mutated porins were found in different lineages. Although most studies are usually focused on carbapenem genes and enzymes, in this work we have shown that other mechanisms are also associated with the resistance phenotype and also need to be controlled.

The AmpC lineage was rapidly substituted by carbapenemase-producing isolates. Two different types of carbapenemases were found in this hospital, OXA-48 and NDM, with some cases of coexistence of *bla*_OXA-48_ and *bla*_NDM_ genes in the same isolates, which is seldom reported worldwide [39, 40]. Several studies have shown that *bla*_OXA-48_, together with *bla*_VIM-1_ and *bla*_KPC_, are the most frequent carbapenemase genes in Spain [41]. Neither *bla*_VIM-1_ nor *bla*_KPC_ were detected in the CHGUV during our sampling period (2015–2018), but they have been reported to have been widespread in Europe at that time [9]. However, we detected two variants of *bla*_NDM_ genes: NDM-1 carbapenemases are less common in Spanish hospitals [42], whereas NDM-23 have never been detected in other Spanish regions, having only been reported in Valencia [33].

After the first detection of the *bla*_OXA-48_ gene in mid-2015, this gene was disseminated through lineages ST307, ST11-KL13 and ST437-OXA48. As previously stated, OXA-48 represents the main carbapenem resistance determinant in Spain, especially in *Klebsiella* spp. strains [41, 43], and has been reported to be widespread throughout Spain and linked to a few successful lineages, including ST11 and ST437 [10, 35]. Thus, we suspect that the interregional dispersion of these clones could be the source of the ST11-OXA-48 and ST437-OXA-48 strains detected in the CHGUV. Regarding the plasmids carrying *bla*_OXA-48_, we observed different variants circulating simultaneously at the CHGUV and among the Spanish isolates (Fig. S4). However, as this plasmid is highly conserved [44], it is difficult to distinguish between clonal expansions or individual acquisitions. Therefore, we cannot confirm whether it was due to a single introduction and later evolution in the hospital, to several introductions, or to a combination of both scenarios. *bla*_NDM_ genes were detected a few months later than *bla*_OXA-48_ and disseminated in lineages ST101 and ST437. The origin of the *bla*_NDM_ genes at the CHGUV could be elucidated thanks to whole-genome analysis.

*bla*_NDM_ genes are usually imported from other regions, associated with individual cases and travel [45]. The emergence of these enzymes in Spain was described in a multiregional study [42 ]. The authors reported two lineages producing *bla*_NDM-1_, ST101 and ST437 that were already present in Valencia. ST101 isolates producing *bla*_NDM-1_ were also reported in Madrid and Catalonia. Epidemiological studies [37] showed that both lineages arrived at the CHGUV through the transfer of a patient from a hospital in Madrid in December 2015 to this hospital in Valencia. This was the time when NDM-related episodes increased in the hospital, mainly associated with the dissemination of the ST101 lineage. Furthermore, we discovered that the *bla*_NDM_ genes found in the CHGUV were carried on two different plasmid backbones, each associated with a different lineage (Fig. 5). A more detailed analysis of the spread of these clones can be found elsewhere [33, 46].

We also found 18 strains without any reported resistance mechanism to carbapenems. However, in most cases, the phenotype was probably related to the presence of *bla*_CTX-M-15_ as reported elsewhere [46].

In summary, we found six lineages comprising the majority of the CRKp population at the CHGUV – ST307, ST11-KL105, ST11-KL13, ST101, ST437-OXA48 and ST437-NDM – disseminating different resistance mechanisms: AmpC, OXA-48, NDM-1 and NDM-23. The SNP diversity within lineages showed that they likely correspond to local clonal expansion, with several cases of possible direct transmission within the hospital (pairwise SNPs <5). All these lineages are globally distributed clones of *K. pneumoniae* and are considered MDR [47]. These four STs have already been identified in Spain as carbapenemase carriers, and they are among the STs widely distributed across Europe [9]. They were related to the acquisition of several antimicrobial-resistant plasmids in different European countries in the late 2000s [48]. For three of the six lineages identified in the CHGUV, we found related samples from other Spanish regions, reflecting the successful dispersion of these lineages, and supporting their interregional spread (Fig. 4). Remarkably, we detected different carbapenemase-coding genes (*bla*_OXA-48_, *bla*_NDM-1_, *bla*_NDM-23_, *bla*_VIM-1_ and *bla*_KPC-3_) within highly related clades that involved both Spanish and CHGUV strains (Figs 4 and S2). This supports the idea that plasmid dissemination is the major factor responsible for the rapid spread of these carbapenemases in the CHGUV and in Spain, as also shown in other studies [49]. It is remarkable how different clones of the same pathogen sharing the same carbapenem resistance mechanisms can have different dissemination dynamics.

It is important to note that the increase in CRKp occurred shortly after 2014, when a large peak of cephalosporin-resistant strains was reported in the CHGUV (up to 45 % of the collected isolates) (Fig. S1). Increased selective pressure, driven largely by the usage of carbapenems to treat infections by cephalosporin-resistant isolates, could explain the rapid increase in CRKp strains. A highly selective environment provides the perfect setting for CRKp lineages to succeed and disseminate.

This work shows the complexity behind the initial colonization of carbapenem-resistant strains in a hospital and remarks on the diversity of the lineages and plasmids involved. Moreover, we demonstrate that most of these lineages were previously present in other regions of Spain, with these probably being the sources of the relevant lineages at the CHGUV. The diversity beyond the increase of non-susceptible strains and the fact that most of the lineages and plasmids arose at the same time highlight the importance of rapid colonization and clone dissemination and plasmid transfer and their effects in the interregional dissemination of CRKp.

## Supplementary Data

Supplementary material 1

Supplementary material 2
