# Supplementary material for: Genomic analysis of the initial dissemination of carbapenem-resistant Klebsiella pneumoniae clones in a tertiary hospital
Source: Microb Genom. 2023 Jun 5;9(6):mgen001032. doi: 10.1099/mgen.0.001032 (PMC10327503; doi:10.1099/mgen.0.001032)
Supplement: Supplementary material 1 [file mgen-9-1032-s001.pdf]

Supplementary figures

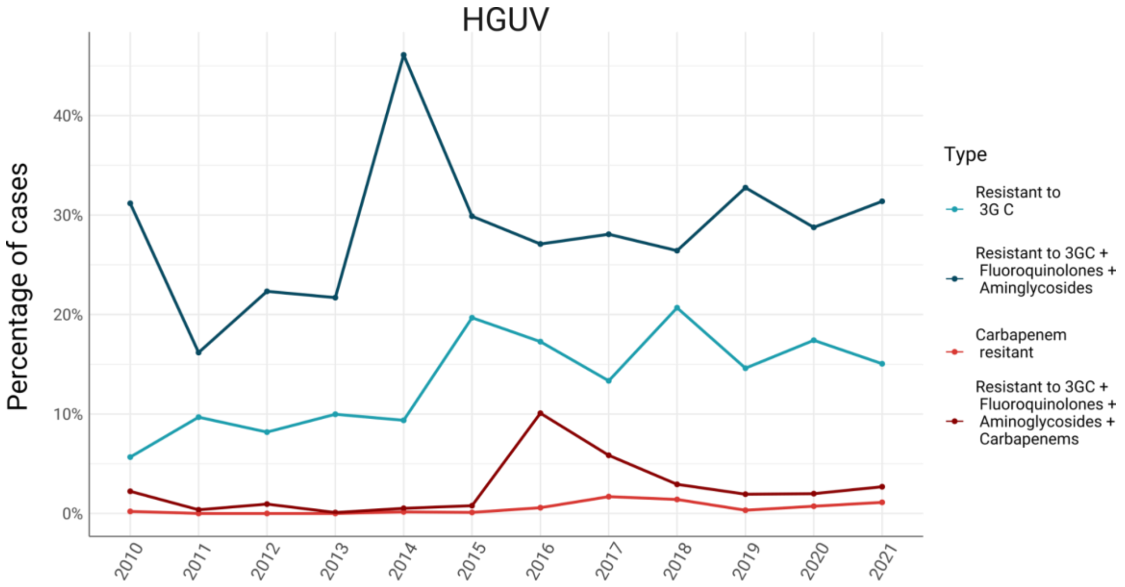

Fig. S1. Relative incidence of cephalosporin- and carbapenem-resistant *Klebsiella pneumoniae* isolates in the CHGUV during the period 2010 to 2021. Data was obtained from the Microbiological Network of the Comunidad Valenciana (RedMIVA).

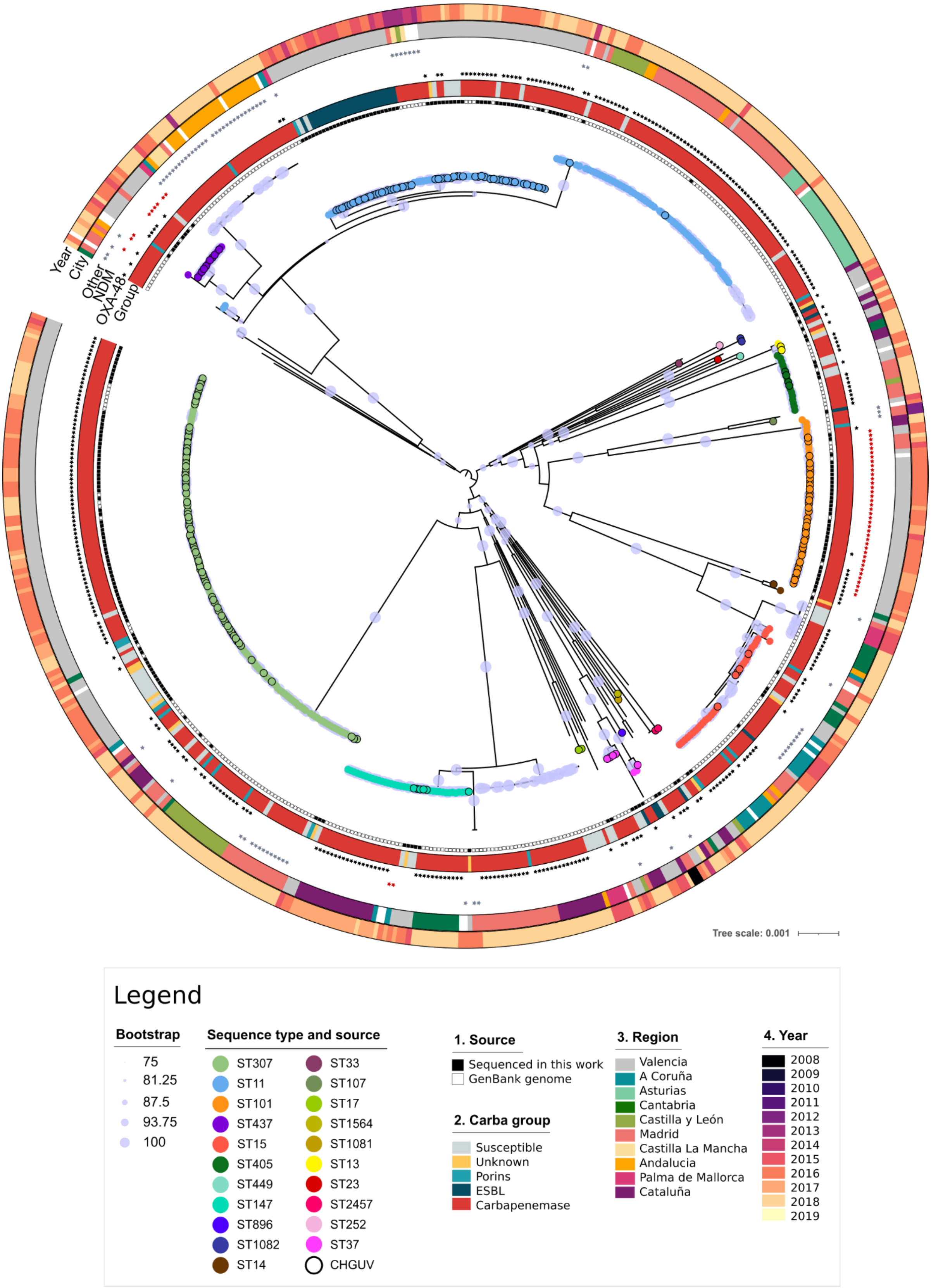

Fig. S2. ML tree of the core genome of the 224 isolates collected in the CHGUV and the 360 Spanish isolates downloaded from GenBank. Dots in the tree indicate the Sequence Type (ST). Only STs found in the CHGUV are shown, and those isolates collected in the CHGUV are encircled in black. Blue dots on the branches represent bootstrap support values. Only values above 75% are indicated.

The innermost ring indicates the source of the isolates, filled in black if they were collected in this work and in grey if obtained from databases. The next ring indicates the carbapenemase groups, whereas the third and the outermost ring indicate the city and year of the collection, respectively.

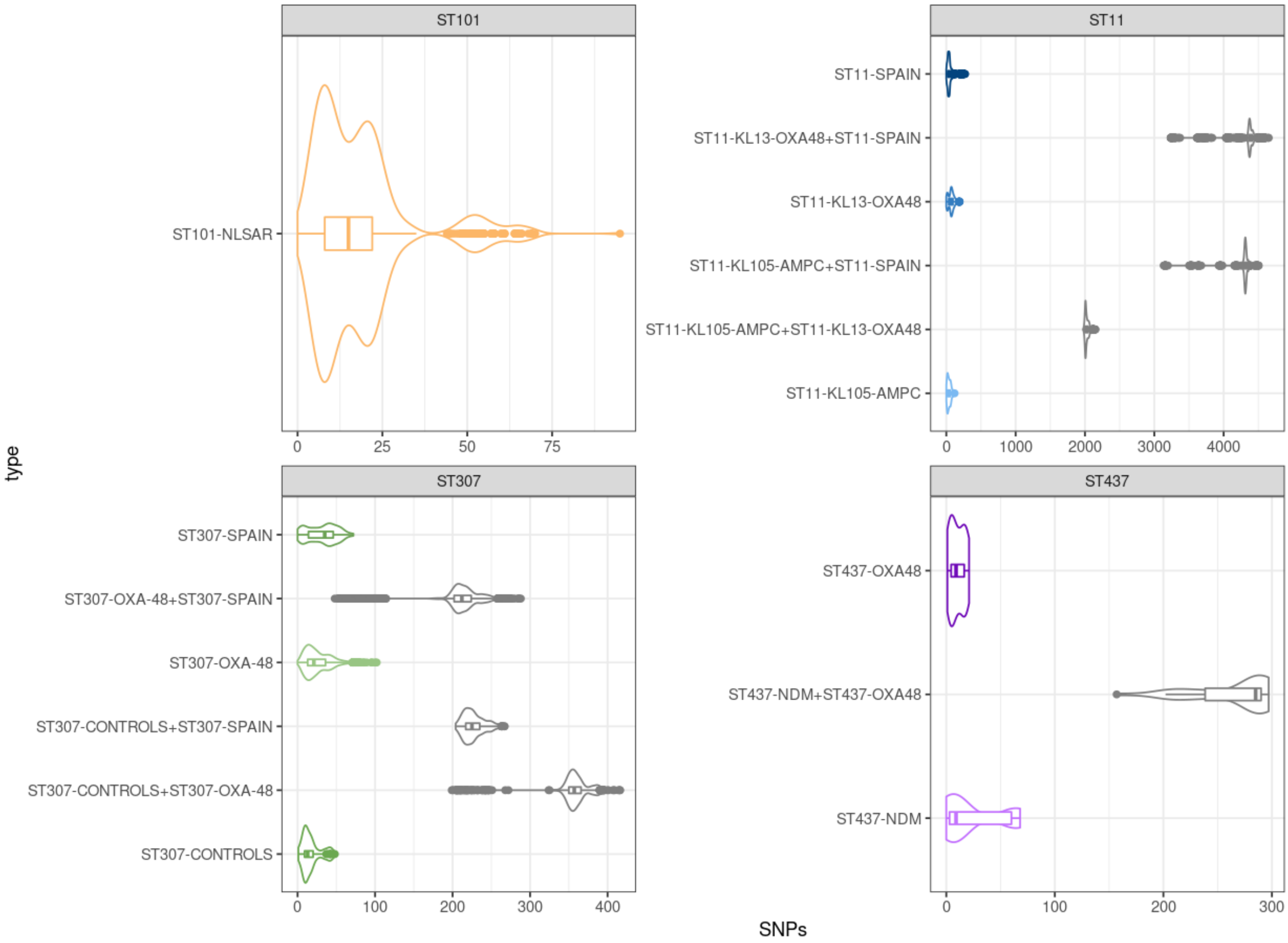

Fig. S3. Distributions of SNP differences within and between lineages of the major STs. SNP distance matrix was obtained from the core genome of each ST.

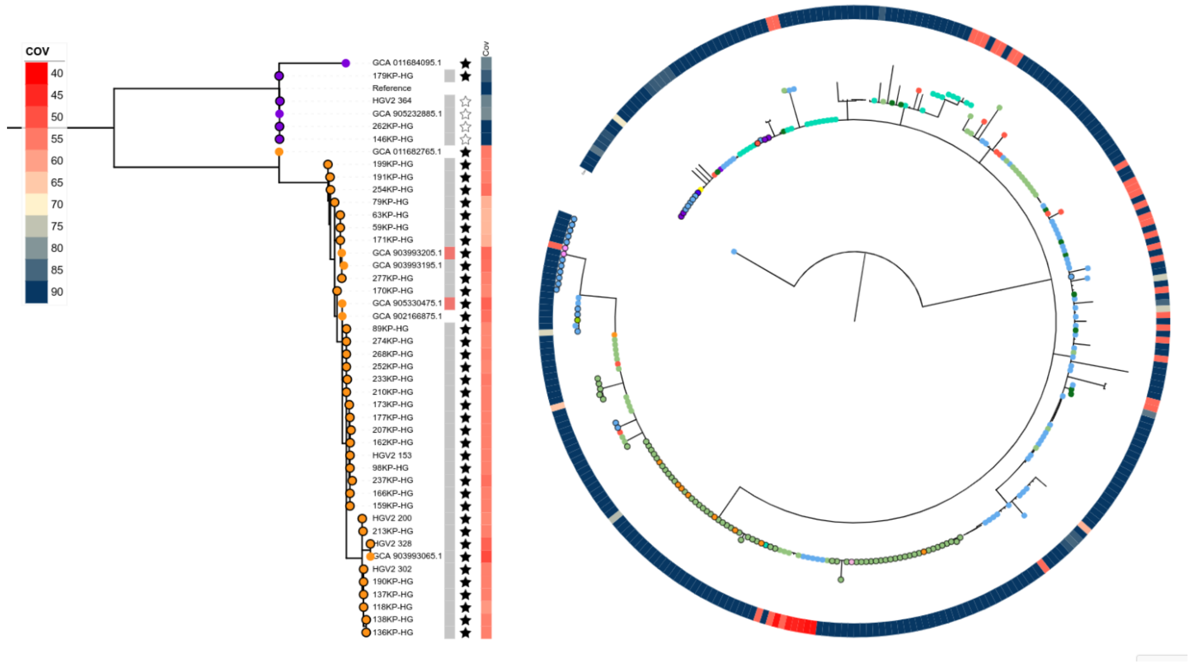

Fig. S4. Phylogenetic trees of OXA-48 and NDM-like plasmids, including the Spanish downloaded genomes. The outer ring represents the coverage value according to the scale at the left side.

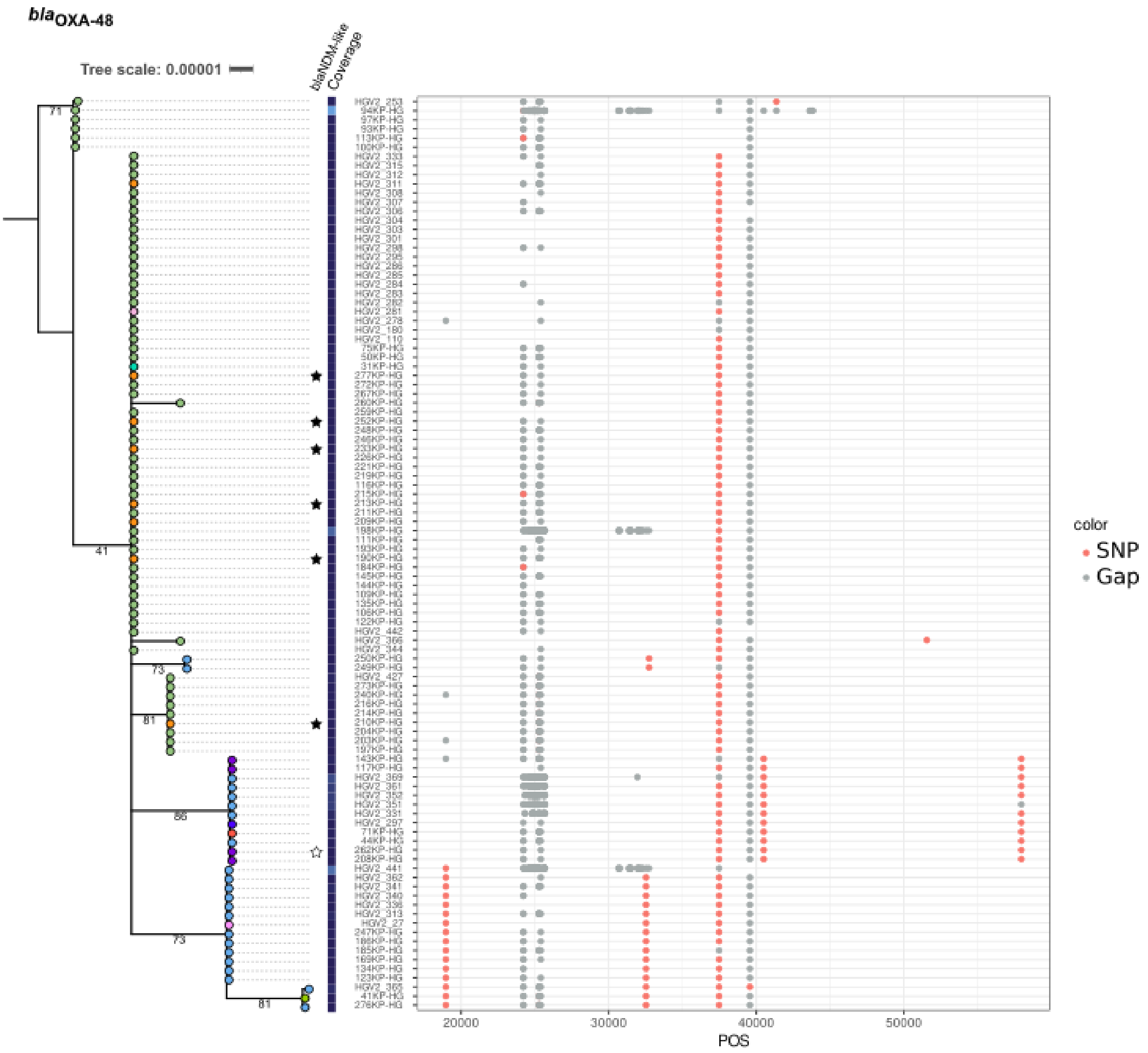

Fig. S5. Maximum likelihood tree of pOXA48 plasmids (61,881 bp) with detail of the SNPs (red dots) in each isolate. Grey dots indicate missing positions in the corresponding sample.

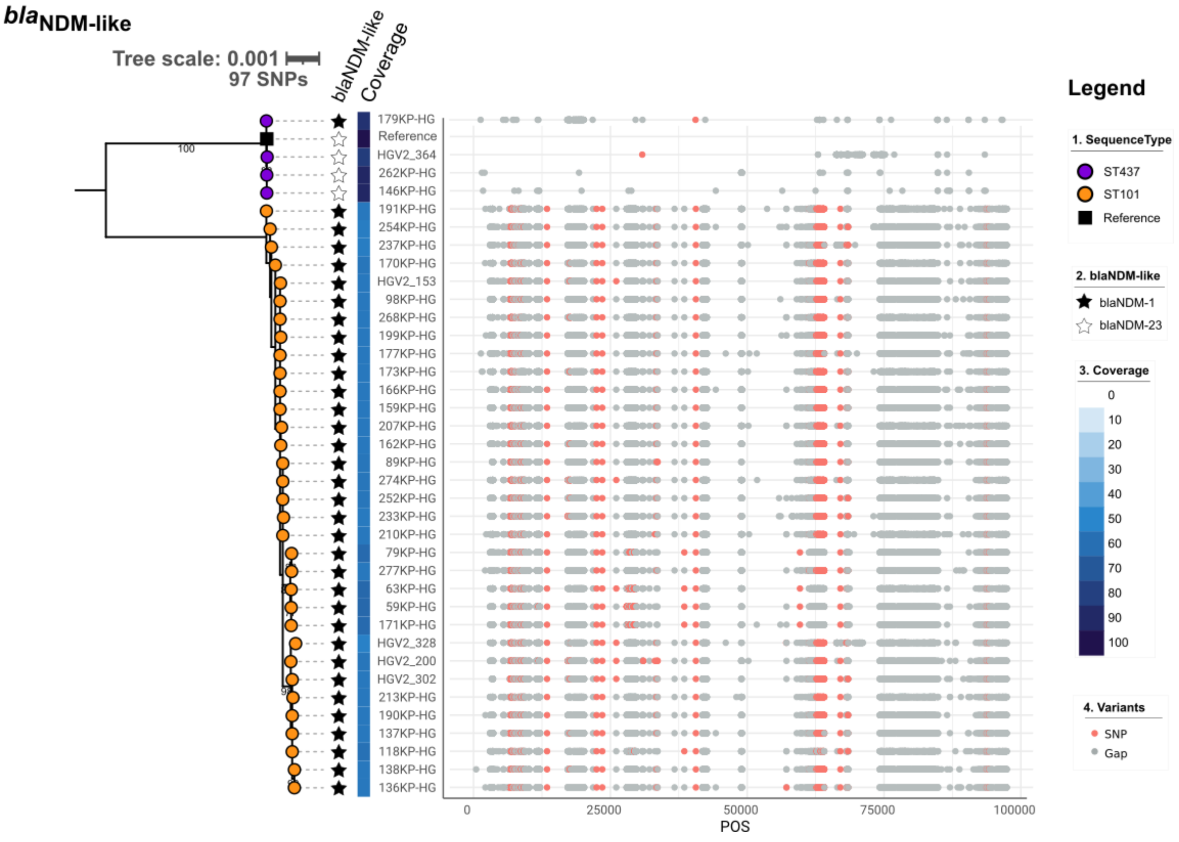

Fig. S6. Maximum likelihood tree of NDM plasmids (97,784 bp) with detail of the SNPs (red dots) in each isolate. Grey dots indicate missing positions in the corresponding sample.

Supplementary Tables

Table S1. Clinical information of the isolates collected and sequenced in this study. The table includes patient data such as gender, age, the strain collection date, and the antimicrobial resistance profile for the antibiotics tested.

Table S2. Genome information of the sequenced isolates, including assembly statistics, carbapenem resistance group, sequence type, capsular type, antimicrobial resistance, and virulence genes. This table includes the accession number of the raw data at SRA.

Table S3. Genome information and metadata for the GenBank Spanish genomes used in this study. It includes information about the isolation source, location, and collection year.

Table S4. Number of gene families included in each core genome and alignment length used to derive the ML phylogenetic trees.

Table S5. Number of SNPs found in ST11, within and between lineages.

Table S6. Number of SNPs found in ST101, within and between lineages.

Table S7. Number of SNPs found in ST437, within and between lineages.

Table S8. Number of SNPs found in ST307, within and between lineages.
